# Network analysis of structural MRI predicts executive function in paediatric traumatic brain injury

**DOI:** 10.1016/j.nicl.2024.103685

**Published:** 2024-10-09

**Authors:** Daniel Griffiths-King, Stefano Seri, Cathy Catroppa, Vicki A. Anderson, Amanda G. Wood

**Affiliations:** a College of Health & Life Sciences & Aston Institute of Health and Neurodevelopment, Aston University, Birmingham B4 7ET, UK; b Department of Clinical Neurophysiology, Birmingham Women’s and Children’s Hospital NHS Foundation Trust, UK; c Brain and Mind Research, Clinical Sciences, Murdoch Children’s Research Institute, Melbourne, Australia; d Department of Psychology, Royal Children’s Hospital, Melbourne, Australia; e School of Psychology, Faculty of Health, Melbourne Burwood Campus, Deakin University, Geelong, Victoria, Australia

**Keywords:** MRI, Traumatic Brain Injury, Development, Morphometry, Morphometric Similarity, Executive Function, Child, Paediatric

## Abstract

**Intro:**

Paediatric traumatic brain injury (pTBI) is likely to result in cognitive impairment, specifically executive dysfunction. Evidence of the neuroanatomical correlates of this executive function (EF) impairment is derived from studies that treat morphometry of brain regions as distinct, independent features, rather than as a complex network of interrelationships. Morphometric similarity captures the *meso*-scale organisation of the cortex as the interrelatedness of multiple macro-architectural features and presents a novel tool with which to investigate the brain post pTBI.

**Methods:**

In a retrospective sample (83 pTBI patients, 33 controls), we estimate morphometric similarity from structural MRI by correlating morphometric features between cortical regions. We compared the *meso*-scale organisation of the cortex between groups then, using partial least squares regression, assessed the predictive validity of morphometric similarity in understanding later executive functioning, two years post-injury.

**Results:**

We found that patients and controls did not differ in terms of the overall magnitude of morphometric similarity. However, a pattern of ROI-level morphometric similarity was predictive of day-to-day EF difficulties reported by parents two years post-injury. This prediction was validated using a leave-one-out, and 20-fold cross-validation approach. Prediction was driven by regions of the prefrontal cortex, typically important for healthy maturation of EF skills in childhood. The *meso*-scale organisation of the cortex also produced more accurate predictions than any one morphometric feature (i.e. cortical thickness or folding index) alone.

**Conclusion:**

We conclude that these methodologies show utility in predicting later executive functioning in this population.

## Highlights

• MRI-based morphometric similarity predicts executive dysfunction in pediatric TBI.
• Fronto-temporal regions predict executive function outcomes post-injury.
• Model outperforms traditional structural MRI measures in prediction accuracy.
• Predictive models can help identify at-risk children, guiding early interventions.

## 1 Introduction

Executive dysfunction is a common and persistent impairment following paediatric traumatic brain injury (pTBI) (Babikian and Asarnow, 2009, Sesma, 2008). Executive functions (EF) are conceptualised of three core skills; working memory, behavioural inhibition and cognitive flexibility (Karr, 2018), from which arise higher-order EFs (i.e. planning and novel problem solving (Krasny-Pacini, 2017). Executive dysfunction seen in children after TBI is evidenced by impairments to performance-based EF measures (Babikian and Asarnow, 2009, Sesma, 2008, Resch, 2019, Urban, 2017, Roncadin, 2004) but also on parent-report measures of higher-order, everyday executive-functioning (Krasny-Pacini, 2017, Mangeot, 2002, Vander Linden, 2019). This ecologically-relevant impairment in daily-living persists over time (Krasny-Pacini, 2017, Keenan, 2018, Vander Linden, 2018) despite improvements on performance-based EF measures (Krasny-Pacini, 2017, Anderson and Catroppa, 2005, Levin, 1997). However, there still exist distinct trajectories of executive dysfunction at the level of individual patients (Anderson and Catroppa, 2005, Catroppa and Anderson, 2009, Polinder, 2015, Konigs, 2018, Ringdahl, 2019). Executive dysfunction has significant links to impairments in the attainment of other, linked skills (for example social skills (Riggs, 2006), potentially predisposing these children to long-term poor developmental outcomes (Perone, S., Almy, B., Zelazo, P.D. Toward an Understanding of the Neural Basis of Executive Function Development, in The Neurobiology of Brain and Behavioral Development, R. Gibb and B. Kolb, Editors., 2018, Gaines and Soper, 2018). Given this, and the importance of early intervention to promote best-attainable outcomes, an important goal for research is to predict those young-people who will experience EF-related difficulties.

Magnetic resonance imaging (MRI) enables us to measure markers of brain health and damage following a TBI (Bigler, 2013). For instance, morphometric analyses of structural T1w MRI (sMRI) show that the brains of children who have experienced a pTBI differ to healthy controls, with evidence of diverging trajectories from typical brain maturation (King, 2019). EFs require intact frontal-striatal circuits (Giedd, 1999, Luciana and Nelson, 1998), with the protracted maturation of this cortical-subcortical network needed for the appropriate unfolding of EF skills over the duration of childhood (Bettcher, 2016). Therefore, EFs are particularly vulnerable to pTBI, because those brain networks are still undergoing maturational changes necessary to subsume these developing skills. MRI markers of these networks should therefore provide insight into the degree of executive dysfunction experienced post-injury, at the individual-level.

Existing literature is inconsistent as to the relationship between sMRI and domains of EF after pTBI. In mild pTBI, thinner cortical thickness of the dorsolateral prefrontal cortex was associated with slower reaction times in a high cognitive load working memory dual-task (Urban, 2017). Smaller parietal and cingulate volumes were related to longer reaction times in a working memory task (Wilde, 2011) and parent-reported working memory problems were significantly associated with the cortical thickness of temporal and parietal ROIs (Merkley, 2008) in pTBI. Whole brain white-matter (WM) volume also predicted long-term inhibition/cognitive flexibility outcomes at 16 years post-injury (Yu, 2018). Despite these findings, others have found no relationship between total grey-matter (GM) volume or frontal pole thickness and performance measures of working memory and cognitive flexibility (Konigs, 2018, Levan, 2016). Changes in volumetric GM are not associated with the improvement of EF following cognitive training (Vander Linden, 2019), and cortical thickness was not significantly associated with parent-reports of executive dysfunction (Vander Linden, 2019). These results highlight the high variability in the purported relationship between indexes of post-injury brain morphometry and EFs.

Methodological factors may account for these inconsistencies, including heterogeneity in cohorts or methodological issues. Existing literature utilises limited sample sizes (range n = 13–23) (Urban, 2017, Vander Linden, 2019, Merkley, 2008, Levan, 2016, Vander Linden, 2019), and either highly reductionist measures of brain morphology across multiple regions/hemispheres (Vander Linden, 2019, Konigs, 2018, Yu, 2018) or a limited number of ROIs (Levan, 2016, Vander Linden, 2019). These factors are likely due to the restricted statistical power with which to test more complex or a greater number of brain regions, given the small sample sizes in the field.

Another key limitation of previous work is the abundance of univariate analyses that treat morphometry of multiple ROIs as independent features, rather than interconnected components of a multivariate whole. A promising technique to establish a link between TBI related changes to the complex organization of morphometry of the brain and later outcomes is morphometric similarity (Seidlitz, 2018). Morphometric similarity networks quantify the biologically meaningful, *meso*-scale organisation of the cortex as a set of statistical similarities between the macro and microstructural properties of all regions of the brain, which can be measured in-vivo, using MRI (Morgan, 2018). Approaching the morphometry of the cortex as a complex network allows us to investigate additional information beyond that of univariate, local approaches to brain structure (Bullmore and Sporns, 2009, Pagani et al., 2016). These approaches have produced meaningful networks of *meso*-scale cortical organisation, which are able to discriminate between controls and patients with autism spectrum disorder (Zheng, 2019), Alzheimer’s disease (Zheng, 2018) and early psychosis (Morgan, 2018). These networks have also successfully been applied to neurodevelopmental cohorts, detecting structural brain (dys)maturation in neonates (Galdi, et al., 2018, Fenchel, et al., 2020), and anatomical disruptions due to regional expression of the abnormal copy number variants (Seidlitz, 2019).

Morphometric similarity may be a powerful tool to understand individual-variance in cognitive abilities. Data has shown a relationship between morphometric similarity and intellectual abilities (IQ) in adolescence (Seidlitz, 2018). However, no previous work has used these networks to focus on specific domains of cognition in paediatrics. In adults, individual differences in morphometric similarity of regions in the prefrontal cortex, were able to predict inhibitory control more accurately than any individual morphometric features (He, 2019). This highlights the potential use of this method in both paediatric populations with pathological changes to the brain and investigating the neuroanatomical basis of executive dysfunction.

Given recent characterisations of TBI as a diffuse disorder of brain connectivity, and the limited findings of local, univariate approaches to brain structure function relationships post-injury, we utilised morphometric similarity as a novel tool with which to investigate executive dysfunction post pTBI. We predicted that, in children who have experienced a pTBI, due to the additive effects of both pathology-related abnormalities and disrupted neural development, that the highly controlled morphometric similarity of cortical regions will be different compared to controls. We also predicted these differences would be primarily found in fronto-temporal regions which are most commonly found to be abnormal following pTBI (King, 2019). We also predicted that quantitative measures of morphometric similarity in patients would be related to later functioning and predict later EF outcomes, beyond any one feature alone.

## 2 Materials and methods

### 2.1 Participants

The data used in the current experiment are a subset of an existing dataset of children who have experienced a TBI between the ages of five and 16 years old. 157 children (patients n = 114) were recruited between 2007 and 2010 into a study on ‘Prevention and Treatment of Social Problems Following TBI in Children and Adolescents’. More detailed descriptions have been published elsewhere (Anderson, 2017, Catroppa, 2017, Anderson, 2013) and utilised by our group in additional secondary analyses (King, 2020, King, 2020, King, 2021). In brief, children with TBI were recruited on presentation to the Melbourne Royal Childrens’ Hospital’s emergency department. Patients were eligible if they: i) were aged between five and 16 years at the time of injury, ii) had recorded evidence of both a closed-head injury and also two post-concussive symptoms (such as headaches, dizziness, nausea, irritability, poor concentration), iii) had sufficient detail within medical records (Glasgow Coma Scale (GCS; (Teasdale and Jennett, 1974), neurological and radiological findings) with which to determine the severity of the injury, iv) had no prior history of neurological or neurodevelopmental disorder, non-accidental injuries or previous TBI, and v) were English speaking. TD controls were also recruited from the community, and were required to meet criteria i), iv) and v). Injury severities were defined clinically using criteria as listed previously (King, 2021).

MRI images were acquired at 3 T on a Siemens Trio scanner (Siemens Medical Systems, Erlangen, Germany) using a 32-channel matrix head coil. The acquisition included a sagittal three-dimensional (3D) MPRAGE [TR = 1900 ms; TE = 2.15 ms; IR prep = 900 ms; parallel imaging factor (GRAPPA) 2; flip angle 9 degrees; BW 200 Hz/Px; 176 slices; resolution 1 × 1 × 1 mm] and sagittal 3D T2-FLAIR non-selective inversion preparation SPACE (Sampling Perfection with Application-optimised Contrast using different flip-angle Evolution) [TR = 6000 ms; TE = 405 ms; inversion time (TI) = 2100 ms; water excitation; GRAPPA Pat2; 176 slices; 1 × 1 × 1 mm resolution matched in alignment to the 3D T1-weighted sequence].

We only included subjects who; a) met strict quality control criteria of Freesurfer outputs (see later), and b) underwent MRI scanning < 90 days post-injury. This resulted in a subset of n = 116 subjects (TBI patients (n = 83) and healthy controls (n = 33)). Group demographics can be seen in Table 1.

**Table 1:** Demographics for patients and controls.

**Group**	**pTBI**	**Controls**	**Comparison**
N	83	33	−
M/F	54/29	20/13	OR = 1.21, p = 0.67^a^
Age at Scanning (median, yrs)	11.07	9.99	F(1,114) = 0.262, p = 0.61^b^
(range, yrs)	6.09–14.82	6.53–15.47	−
Age at Injury (median, yrs)	10.92	NA	−
(range, yrs)	5.92–14.67	NA	−
Injury-Scan Interval (median, days)	34	NA	−
(range, days)	1–88	NA	−
**Injury Severity**			
Mild	47	NA	−
Moderate/Severe c	36	NA	−

a Fisher’s exact test (OR = odds-ratio),

b One-Way ANOVA,

c Mild Complicated TBI + Moderate TBI + Severe TBI.

### 2.2 MRI processing

3D tissue segmentation and estimation of brain morphometry from T_1_-weighted (T_1_w) MR images were conducted using an established pipeline (Freesurfer version 6.0; see Fischl (Fischl, 2012) for review). The steps involved are documented elsewhere (Fischl, 2004) but briefly, T_1_w images were stripped of non-brain tissues (Segonne, 2004), GM/WM boundaries were tessellated and topology was automatically corrected (Segonne et al., 2007, Fischl et al., 2001). Finally, deformation of this surface was performed, to optimally define tissue boundaries using intensity gradients (Dale and Sereno, 1993, Dale et al., 1999, Fischl and Dale, 2000). Where available, 3D T_2_-weighted (T_2_w) FLAIR MRI were used to refine the boundary between the pial surface and dura within the Freesurfer algorithm, to good effect. In this study, Freesurfer was used to estimate multiple morphometric features for 34 regions-of-interest (ROIs) per hemisphere, based upon the cortical parcellation of the Desikan-Killiany atlas (Desikan, 2006). This parcellation was chosen over a more fine-grained parcellation scheme due to limitations of statistical power if a greater number of ROIs were analysed. Using Freesurfer, we calculated seven metrics with which to estimate the morphometry and shape of the cortex. This included surface area, curvature index, folding index, gaussian curvature, mean curvature, cortical thickness, and cortical volume. The quality of Freesurfer outputs was assessed using Qoala-T (Klapwijk, 2019), a decision support tool to guide systematic selection of cases requiring manual editing. Multiple cases within the original TBI cohort also had frank parenchymal lesions to the grey matter ribbon. For these cases, Freesurfer has limited applicability with its standard processing pipeline and thus an adjusted pipeline was utilised as used in our previous studies of this dataset (King, 2021). Eight cases (with lesions) were retained for analysis using this pipeline.

### 2.3 Morphometric similarity

Previously morphometric similarity was estimated from morphometric features measured in-vivo by both structural and diffusion MRI (Seidlitz, 2018). However, we highlighted significant correspondence between this morphometric similarity and that estimated with only features obtainable from a T1w MRI (King and Wood, 2020) and recent papers have similarly adopted this T1w-only approach (He, 2019), as does the current study.

To estimate morphometric similarity, the nodes for network construction were the ROIs from the Desikan-Killany atlas. At an individual-level, the seven morphometric features estimated for each node can be expressed as a set of n vectors of length 7, with each vector as a different anatomical region (n = 68), and each element of the vector a different morphometric measure. To normalize measures within this length 7 vector, each morphometric feature is demeaned and SD scaled across the 68 regions, using Z-scores. A correlation matrix (pearson correlation) was generated for each participant, where each element of the matrix is the correlation between the feature vectors for every possible pairwise combination of regions. This correlation matrix represents the morphometric similarity derived *meso*-scale cortical organisation for each participant. These were thresholded across multiple network densities (*x* = 5 to 40 in increments of 5), retaining only *x*% strongest absolute values of morphometric similarity across the graph. This has the effect of removing potential false-positive estimates of morphometric similarity. We also investigated the unthresholded graph.

For each node/ROI, we calculated both nodal degree and nodal strength. Nodal degree was the number of edges that had survived thresholding for each node. Normalised nodal strength was calculated as the ‘magnitude’ of morphometric similarity for each node. This is defined as the sum of the MS weights of all of the edges of node *i* (Fornito, A., Zalesky, A., Bullmore, E. Fundamentals of Brain Network Analysis., 2016), normalised by the degree of the node (nodes with a higher number of edges will by definition have a greater magnitude of morphometric similarity). We also calculated the average nodal strength across the network to provide a global measure of the magnitude of morphometric similarity.

### 2.4 Neuropsychology Assessment: Executive function

EFs were assessed for all participants at approximately 24-months post-recruitment (or post-injury for pTBI group, M(SD) = 754(80) days post-injury) using performance-based neuropsychological testing. Several age-appropriate neuropsychological tests were administered to participants to index EF skills, and these were from three typical, age-appropriate test batteries, to represent components of a three-factor EF model (Diamond, 2013); i) Set-shifting: Creature Counting, Tests of Everyday Attention – Children (TEA-Ch (Manly, et al., 1999), ii) Inhibition: Color-Word Interference, Delis-Kaplan Executive Function System (D-KEFS (Delis et al., 2001), and Walk Don’t Walk and Sky Search (TEA-Ch (Manly, et al., 1999) and iii) Working Memory: Digits backwards, Wechsler Intelligence Scale for Children (WISC-IV (Wechsler, 2003). These measures were selected from a wider battery of administered neuropsychological tests as part of the wider study. (Table 2). As per Diamond (Diamond, 2013), we included a measure of selective-attention in the domain of inhibition, with evidence highlighting the high correlation of this skill with other EF domains (Santa-Cruz and Rosas, 2017, Downing, 2015). Performance scores for the neuropsychological test batteries were converted to age-scaled scores (M = 10, SD = 3). To provide a summary score for common EF performance, we summed these age-scaled scores across subtests whereby higher scores equalled better performance (reverse scoring where necessary). The Global Executive Composite T-score (GEC; M = 50, SD = 10), from the Behavior Rating Inventory of Executive Function (BRIEF (Gioia, 2000) was completed by parents as a measure of their child’s EF in daily-living with (higher scores = greater difficulties). Fifty-nine participants had completed neuropsychological testing to calculate EF summary scores and 59 (subsets not identical) participants had BRIEF-GEC scores available.

**Table 2:** Neuropsychological tests and subtests used to group patients on executive functioning outcome 2 years post-injury.

**EF Domain**	**Battery**	**Subtest**	**Measure**
Set Shifting	TEA-Ch	Creature counting	Accuracy (no. correct)
	TEA-Ch	Creature counting	Time taken
Inhibition	D-KEFS	Color-word interference – condition 3	Time Taken
	D-KEFS	Color-word interference – condition 4	Time Taken
	TEA-Ch	Walk-don’t-walk	Score
	TEA-Ch	Skysearch	Attention Score
Working Memory	WISC-IV	Digit span backwards	Score

### 2.5 Statistical analyses

#### 2.5.1 Between-group differences in the magnitude of morphometric similarity

Firstly, we investigated differences in the magnitude of morphometric similarity between patients and controls. For each threshold, we conducted a GLM to test the effect of group (TBI vs Controls) on average nodal strength (graph-level), whilst controlling for age at scanning, sex, age × sex, and estimated total intracranial volume (eTIV). This was then repeated for all ROIs of the unthresholded network to investigate the effect of group on nodal strength. For all GLM analyses, the t-statistic for the estimated effect of group on was used to estimate the effect size using Hedge’s g (reference) corrected for unequal sample sizes (Rosnow et al., 2000). All p-values reported are FDR-corrected (Benjamini and Hochberg, 1995) across the number of thresholds or the number of ROIs respectivey.

#### 2.5.2 Predicting EF outcome using MS in pTBI

To assess whether morphometric similarity was related to later function EF outcomes in the patient group, we utilised a supervised learning approach using partial least squares regression (‘plsRglm’ package in R (Bertrand and Maumy-Bertrand, 2018). This multivariate approach finds the maximal low-dimensional covariance between components derived from a high-dimensional set of predictors (in this case morphometric similarity across ROIs) and a univariate response (either EF score or BRIEF). This approach is commonly used when the number of predictors exceeds the number of observations (Krishnan, 2011) and has previously been used to examine the relationship of brain structural changes and behaviour (Phan, 2010).

Firstly, we decompose the predictor variables into latent variables (components) which simultaneously model the predictors and predict the response variable (Krishnan, 2011). The predictor matrix consisted of either the degree or normalised strength of each node of the morphometric similarity network, for each participant. Using a linear model, the potential confounding effect of eTIV, age, sex and age × sex interaction was regressed out of values for nodal degree/strength. Then, at each network threshold, a PLS regression model was fitted between components derived from the resultant predictor matrix and the outcome variable. Components were derived and the number of components to retain in the final model was decided based upon the local minima of AIC, calculated using corrected degrees of freedom of the model (Krämer and Sugiyama, 2011). Given the model with the retained number of components, we report the predicted R^2^, Pearson correlation coefficient and mean absolute error (MAE) between actual and predicted response scores. Standard linear regression R^2^ is a biased estimate of model performance (Scheinost, 2019), whereas predicted R^2^ is more appropriate for quantifying regression accuracy (Poldrack et al., 2019), calculated as;

(1) $$\mathrm{Predicted}R^{2}=1-NormalisedMSE$$

where the normalised MSE (Mean Squared Error) is equal to;

(2) $$\mathrm{Predicted}R^{2}=1-\frac{\mathrm{MSE}(PredictedValue,ObservedValue)}{\mathrm{MSE}(ObservedValue,MeanValue)}$$

Therefore, all references to R^2^ from here relate to predicted R^2^. MAE is also a useful measure for quantifying regression accuracy, especially for prediction of standardised measures such as the BRIEF as this represents error in the units of the given measure (Poldrack et al., 2019).

We also calculate bias corrected and accelerated bootstrapped (Bastien et al., 2005) (1000 bootstraps) weightings of each predictor, assessing which brain regions most consistently load onto the components and are contributing most to the explanation of variance in the model.

To validate the model, leave-one-out cross validation (LOO-CV) was used to produce a numerical prediction of outcome for each participant independent of the supervised learning procedure. The PLS regression was applied once for each patient, using all other patients as a training set and using the selected patient as a single-case test set. The predictive accuracy was again assessed with predicted R^2^, Pearson correlation coefficient and MAE. This LOO-CV approach was selected due to the limited sample size (LOO-CV enables us to maximise training data) however, LOO-CV approaches can be overfit to the dataset, resulting in high variance and less generalizability. To validate our findings, we repeated the testing using cross-fold validation. Specifically, we used 20-fold validation. In these approaches, the dataset is divided into n folds, with n-1 folds used for training and the remaining fold used for testing. Given that multiple possible iterations of these folds exist, we shuffle the folds for 100 repetitions, and calculate mean prediction R^2^, the 95 % confidence interval of this mean, and median prediction R^2^.

Subsequently, we conducted similar predictions using the individual morphometric features at each region as the predictors, to determine whether morphometric similarity provided greater information for prediction than the features alone.

### 2.6 Data availability

#### 2.6.1 Participant data

This cohort were originally recruited for a study that had previously received ethical approval via the Human Research and Ethics Committee of Royal Children’s Hospital, Melbourne, Australia. Written informed consent to participate in the original study was provided by the participants' legal guardian/next of kin of enrolment to the study. A favourable opinion was granted by Aston University Ethics Committee for the secondary analysis of the TBI datasets.

Data from the TBI cohort was obtained for secondary analysis in the current study from study authors via a Material Transfer Agreement between the Murdoch Children’s Research Institute & Aston University. The data that support the findings of this study are available on request from the corresponding author. The data are not publicly available due to privacy or ethical restrictions, consent was not provided for public sharing at the time.

#### 2.6.2 Code

The current methods used a number of third party, open-access software and tools. All analyses were conducted using R (v3.3.2) (Krasny-Pacini, 2017), in RStudio (v2023.06.1.524) (Resch, 2019). Descriptive statistics (and subsequent comparisons) were conducted using the “stats” package from core R (Krasny-Pacini, 2017), and “psych” (v2.4.6) package (Urban, 2017). Morphometric similarity was estimated using commands from “brainGraph“ (v2.7.0) (Levin, 1997). Controlling for additional variables, and comparisons between groups of morphometric similarity were conducted using “Car” (v3.1–2) and “Effects” (v4.2–2) packages (Roncadin, 2004, Mangeot, 2002, Vander Linden, 2019). Plots were generated using “ggplot2” (v3.5.1) (Keenan, 2018), “ggcorrplot” (v0.1.4.1) (Vander Linden, 2018) and “ggExtra” (v0.10.1) (Anderson and Catroppa, 2005). PLS analyses, and cross-validation was conducted using “plsRglm” (v1.5.1) (Catroppa and Anderson, 2009, Polinder, 2015).

## 3 Results

### 3.1 Patient-control differences in the magnitude of morphometric similarity

Comparing pTBI patients against controls, mean difference in the magnitude of morphometric similarity (adjusted for age at scanning, sex, age × sex, and eTIV) across the brain was not significant, across all thresholds tested (all p_fdr_ > 0.05). This non-significant difference was also reported when looking at the nodal strength across the 68 regions in the unthresholded network (See supplementary materials).

These lack of significant differences were potentially due to the inhomogeneity of the patient group and thus we conducted exploratory, post-hoc analyses of multiple patient-control differences, specifically across groups of clinically relevant EF impairment and injury severity groups. Still, no significant differences were found (See supplementary materials).

### 3.2 Predicting EF outcome using morphometric similarity in pTBI

There was a range in EF outcomes, for both EF summary scores (M = 70, SD = 12, range = 34 – 97) and BRIEF GEC (M = 48, SD = 11, range = 35 – 83) across the pTBI group. We conducted PLS regression for each outcome to predict these scores from the ROI-level magnitude of morphometric similarity estimated across all nine density thresholds tested.

For EF summary scores, across all thresholds, the AIC indicated the retention of zero predictor components, thus no models were generated. This suggests that morphometric similarity is unrelated to later performance-based EF in this sample.

**Fig. 1:**
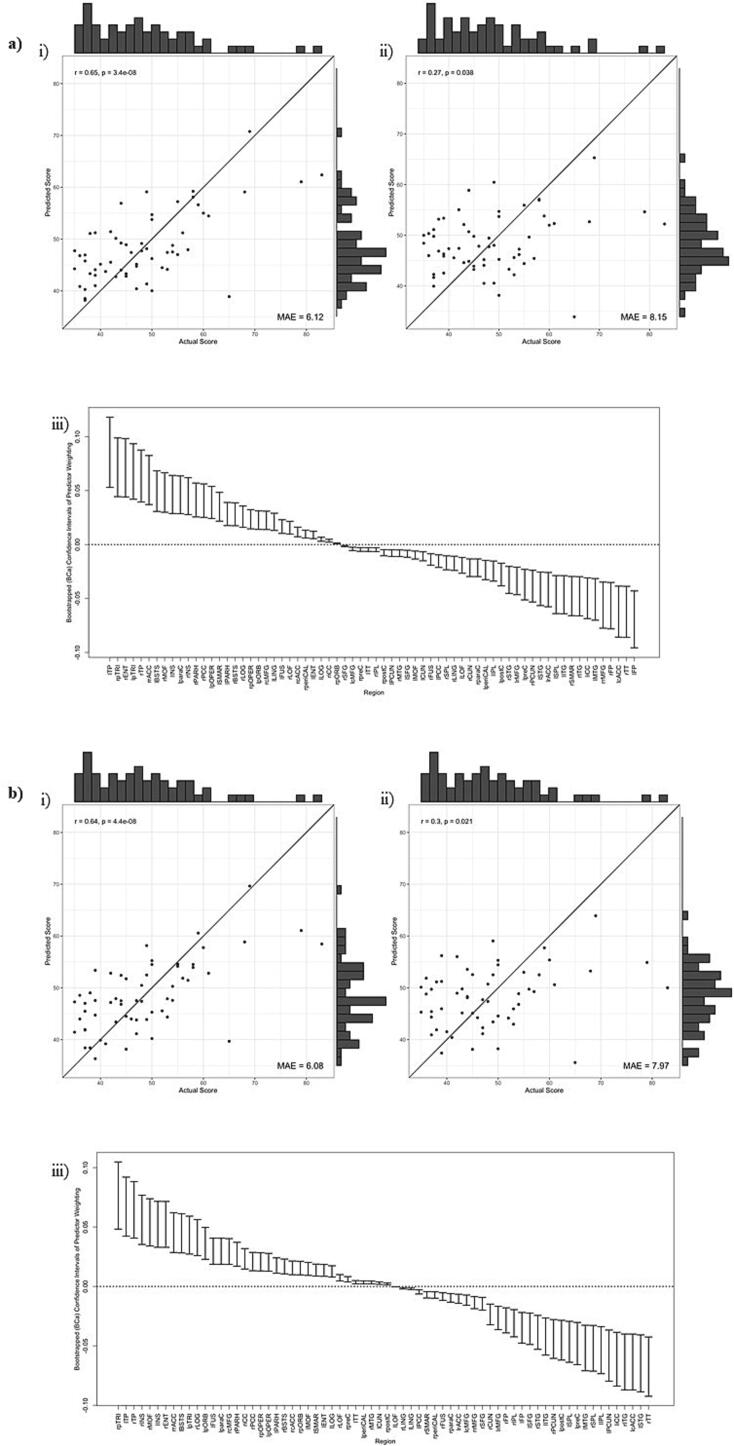

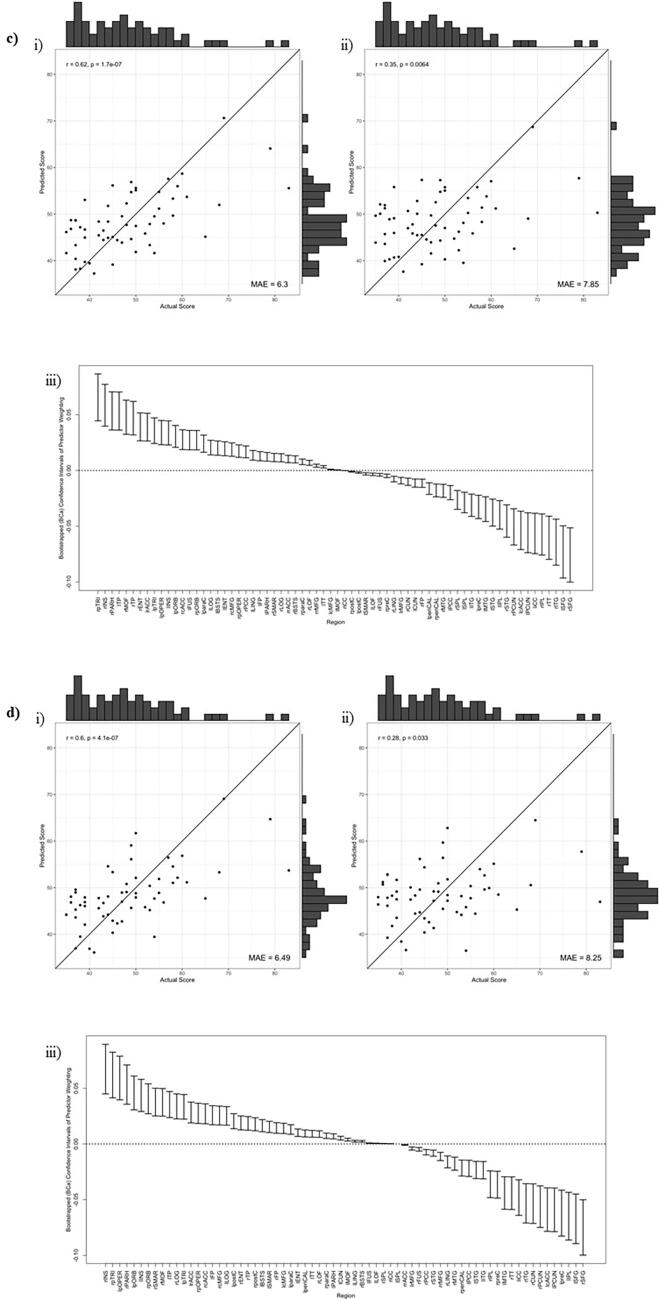
**Results of PLS regression**. Results are shown across network density thresholds as follows; a) 0.25, b) 0.30, c) 0.35, d) 0.40. **i)** The correlation between actual and predicted BRIEF scores based upon the PLS regression model, **ii)** the correlation between actual and predicted BRIEF scores based upon leave-one-out cross-validation of the PLS regression model. For **i)** and **ii)** the line represents x  = y, indicative of perfect prediction of a model. **iii)** The bootstrapped (bias-corrected and accelerated) CI for PLS weightings for each ROI.

However, for BRIEF scores, AIC suggested retention of one component, across network densities of 0.25, 0.30, 0.35, & 0.40. In these models, highly significant positive correlations were found between actual and predicted BRIEF scores, with the PLS models explaining around 40 % variance in BRIEF scores (R^2^ = 0.36 − 0.42), with a relatively low error between predicted and actual scores (MAE = 6.08–6.49). When validated using a leave-one-out cross-validation (LOO-CV) approach, there were significant positive correlations between predicted and actual outcomes (see Table 3) and error was still low (MAE = 7.85–8.25), however the variance explained by the model was much lower (as expected), specifically peaking at around 8 %. Fig. 1 highlights these predictions graphically.

**Table 3:** Description of models where components were retained when predicting executive dysfunction from the morphometric similarity of those following a pTBI.

**Outcome**	**Graph Metric**	**Network Density**	**N. Comp.**	**Full Dataset**			**LOO-CV**		
				**R^2^**	**r(y_pred._, y_actual_)**	**MAE**	**R^2^**	**r(y_pred._, y_actual_)**	**MAE**
BRIEF-GEC	Degree	0.25	1	0.42	r = 0.65, p < 0.001	6.12	0>.01	r = 0.27, p = 0.038	8.15
		0.30	1	0.41	r = 0.64, p < 0.001	6.08	0.03	r = 0.30, p = 0.021	7.97
		0.35	1	0.38	r = 0.62, p < 0.001	6.3	0.08	r = 0.35, p = 0.006	7.85
		0.40	1	0.36	r = 0.60, p < 0.001	6.49	0.01	r = 0.28, p = 0.033	8.25

**N. Comp.** = Number of components retained in the model, **LOO-CV** = Leave-one-out cross-validation, **R^2^** = Predicted R^2^, **r** = Pearson Correlation Coefficient, **MAE** = Mean Absolute Error between predicted values and actual values.

Cross-validation analyses found that the model derived from the degree of nodes according to morphometric similarity (specifically thresholded at 0.35) was stable when tested using LOO-CV or 20-fold CV (Table 4). This, plus the narrow 95 % CI of mean R^2^ performance across the 100 repetitions of the cross-fold validation, indicates that this model is robust to these changes in size of training set, and the specific data points included in training.

**Table 4:** Performance of models when predicting executive dysfunction from the morphometric similarity of those following a pTBI. Performance is tested using both “Leave-one-out” and “20-fold” cross-validation approaches. Feature sets resulting in negative R^2^ or resulting in no components being retained are not reported. The best performing model is highlighted.

**Outcome**	**Graph Metric**	**Network Density**	**N. Comp.**	**LOO-CV**			**20-Fold CV**		
				**R^2^**	**r(y_pred._, y_actual_)**	**MAE**	**Mean R^2^**	**95 % CI Mean R^2^**	**Median R^2^**
BRIEF-GEC	Degree	0.30	1	0.03	r = 0.30, p = 0.021	7.97	0.03	(0.02 − 0.03)	0.03
		0.35	1	0.08	r = 0.35, p = 0.006	7.85	0.08	(0.07 − 0.08)	0.08
		0.40	1	0.01	r = 0.28, p = 0.033	8.25	0.00	(0.00 − 0.01)	0.00

**N. Comp.** = Number of components retained in the model, **LOO-CV** = Leave-one-out cross-validation, **20-Fold CV** = Cross validation over 20-folds of the data (i.e. using 95 % of data for training and 5 % for testing), repeated over 100 random sets of 20-folds, **R^2^** = Predicted R^2^, **r** = Pearson Correlation Coefficient, **MAE** = Mean Absolute Error between predicted values and actual values.

**Fig. 2:**
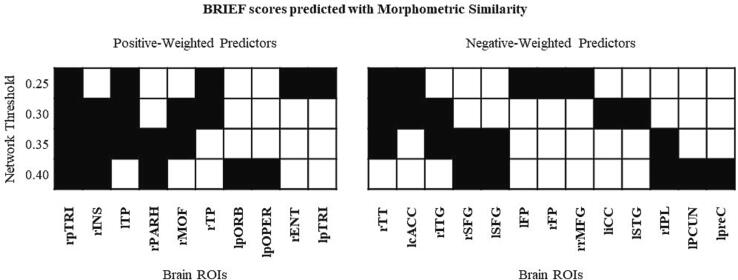
**Predictors of executive function.** Plot of top five positive/negative weighted predictors (degree of ROIs) of BRIEF GEC across network thresholds from 0.25 density to 0.40 where black squares indicate relevant predictors. (*r = right hemisphere, l = left hemisphere, pTRI = pars triangularis, INS = r insula, TP = temporal pole, PARH = parahippocampal gyrus, MOF = middle orbitofrontal, pORB = pars orbitalis, pOPER = pars opercularis, ENT = entorhinal, TT = transverse temporal, cACC = caudal anterior cingulate, ITG = inferior temporal gyrus, SFG = superior frontal gyrus, FP = frontal pole, rMFG = rostral middle frontal gyrus, iCC = isthmus cingulate, STG = superior temporal gyrus, IPL = inferior parietal lobule, PCUN = precuneus, preC = precentral sulcus*).

Bootstrapped predictor weightings showed that the CIs appeared narrow but some overlap between regions was present. Fig. 2 illustrates, across network densities, a consistent set of regions most-highly weighted by components, therefore explaining the greatest proportion of variance in BRIEF outcome.

### 3.3 Predicting EF outcome using individual morphometric features in pTBI

Table 5 shows the results of PLS models using individual morphometric features to predict both EF summary and BRIEF GEC scores. Unlike for morphometric similarity, PLS models were generated for curvature index, Gaussian curvature and cortical volume in predicting EF score. Model performance varied across the structural features based on LOO-CV across evaluation metrics (R^2^ = -0.55 − 0.04, MAE = 9.67–––10.56). The negative values for predicted R^2^ suggest an overfit model which performs poorly on training data. In predicting BRIEF-GEC curvature index, folding index, gaussian curvature, cortical thickness and cortical volume models explained less variance than morphometric similarity models, with weaker correlations between observed and predicted outcomes and slightly higher MAE (R^2^ = 0.10 − 0.19, MAE = 6.99 – 7.61, See Table 5 for more details).

**Table 5:** Description of models where components were retained when predicting executive dysfunction from individual morphometric features of those following a pTBI.

**Outcome**	**Morphometric Feature**	**N. Comp.**	**Full Dataset**			**LOO-CV**		
			**R^2^**	**r(y_pred._, y_actual_)**	**MAE**	**R^2^**	**r(y_pred._, y_actual_)**	**MAE**
EF Score	Curvature index	1	0.17	r = 0.41, p = 0.001	8.71	−0.55	r = -0.37, p = 0.004	10.56
	Gaussian Curvature	1	0.17	r = 0.41, p = 0.001	8.98	0.04	r = 0.24, p = 0.066	9.67
	Cortical Volume	1	0.20	r = 0.45, p < 0.001	8.79	0.01	r = 0.23, p = 0.073	9.79
BRIEF-GEC	Curvature index	1	0.14	r = 0.37, p = 0.004	7.61	−0.19	r = -0.21, p = 0.11	8.62
	Folding Index	1	0.18	r = 0.43, p < 0.001	7.48	−0.22	r = -0.16, p = 0.22	8.78
	Gaussian Curvature	1	0.15	r = 0.39, p = 0.002	7.50	0.01	r = 0.20, p = 0.14	8.09
	Cortical Thickness	1	0.10	r = 0.32, p = 0.013	7.57	−0.02	r = 0.14, p = 0.30	8.03
	Cortical Volume	1	0.19	r = 0.43, p < 0.001	6.99	−0.05	r = 0.17, p = 0.20	7.82

**N. Comp.** = Number of components retained in the model, **LOO-CV** = Leave-one-out cross-validation, **R^2^** = Predicted R^2^, **r** = Pearson Correlation Coefficient, **MAE** = Mean Absolute Error between predicted values and actual values.

The LOO-CV generated predictions from these models also explained less variance than the morphometric similarity model, with the majority of predicted R^2^ values being negative (R^2^ = -0.19 − 0.01). It is important to note, that conducting cross-fold validation on the models which used individual morphometric features as input, revealed increased performance in the Gaussian curvature model (see Table 6), prediction R^2^ increased from R2 = 0.01 when tested using LOO-CV to mean R^2^ = 0.11 (95 % CI [.11 − 0.11]) when tested using cross-fold validation (20-fold). Whilst this may indicate an improvement to a performance greater than that of the morphometric similarity model (R2 = 0.11 vs. R2 = 0.08 respectively), it may also indicate that the Gaussian Curvature model is highly susceptible to outliers in the data, struggling to perform well in outlier cases when they are the only instance being tested on (i.e. in the LOO-CV scenario), resulting in a much reduced R^2^ for the LOO-CV approach.

**Table 6:** Performance of models when predicting executive dysfunction from individual morphometric features of those following a pTBI. Performance is tested using both “Leave-one-out” and “20-fold” cross-validation approaches. Feature sets resulting in negative R^2^ or resulting in no components being retained are not reported. The best performing model is highlighted.

**Outcome**	**Morphometric Feature**	**N. Comp.**	**LOO-CV**			**20-Fold CV**		
			**R^2^**	**r(y_pred._, y_actual_)**	**MAE**	**Mean R^2^**	**95 % CI Mean R^2^**	**Median R^2^**
EF Score	Curvature index	1	−0.55	r = -0.37, p = 0.004	10.56	0.06	(0.06 − 0.07)	0.07
	Gaussian Curvature	1	0.04	r = 0.24, p = 0.066	9.67	0.07	(0.06 − 0.07)	0.07
BRIEF-GEC	Curvature index	1	−0.19	r = -0.21, p = 0.11	8.62	0.06	(0.05 − 0.08)	0.08
	Gaussian Curvature	1	0.01	r = 0.20, p = 0.14	8.09	0.11	(0.11 − 0.11)	0.11

**N. Comp.** = Number of components retained in the model, **LOO-CV** = Leave-one-out cross-validation, **20-Fold CV** = Cross validation over 20-folds of the data (i.e. using 95 % of data for training and 5 % for testing), repeated over 100 random sets of 20-folds, **R^2^** = Predicted R^2^, **r** = Pearson Correlation Coefficient, **MAE =** Mean Absolute Error between predicted values and actual values.

The bootstrapped CIs for regional predictors for all individual features were much wider than those in the morphometric similarity PLS model (see supplementary materials).

## 4 Discussion

### 4.1 Patient-control differences in the magnitude of morphometric similarity

The current study adopts a morphometric similarity network approach (Seidlitz, 2018) to the investigation of the neuroanatomical correlates of later executive dysfunction following pTBI. We found no differences, across any of the morphometric similarity network thresholds tested. This was true at both the graph and ROI-level, between pTBI patients and controls. For these analyses, the entire pTBI group were considered as a single, homogenous group. However, we know there are large heterogeneities in terms of structural damage post-pTBI (King, 2019), and in EF outcomes (Ringdahl, 2019). In a previous study of the current dataset, we also showed that structural covariance derived from only cortical thickness measures, differed between patients and controls when patients where stratified based on an EF impairment rule (Beauchamp, 2015), with differences only being seen in those patients showing an impairment in EF (King, et al., 2021). Therefore, we compared morphometric similarity between groups derived from those with clinically relevant impairment or groups based on injury severity. We still found no significant group differences in the magnitude of morphometric similarity averaged across the cortex. However, although we did not find differences in morphometric similarity between groups, it is important to note the unequal sample sizes which are present between the two groups, which could have the effect of reducing capacity to detect significant effects.

### 4.2 Predicting EF outcome using morphometric similarity in pTBI

Despite finding no group differences in the magnitude of morphometric similarity, we were able to predict later executive dysfunction in pTBI patients. We built models that predicted BRIEF – GEC from the nodal degree of ROIs – specifically most accurately at 35 % density. Whilst arbitrary, this threshold may balance the inclusion of sufficient data (at lower network densities the network would become sparse and contain less information) with the control of false positive edges (at higher densities weak, less stable edge weights are included). Future research should consider the potential of identifying thresholds (or thresholding methodologies) that consistently explain greater variance in cognitive abilities.

Whilst training models performed moderately well, predicted R^2^ values suggested that our model successfully explained/predicted only 8 % of the variation in parent-report EFs in cross-validation (at 35 % density). This represents a significant drop compared to the training data, potentially indicating limited generalisability of the model. Whilst the cross-validated performance represents a small proportion of variance, we would posit that this represents a model which could be used for meaningful prediction. Firstly, this result is in the context of both LOO-CV and 20-fold cross-validation sample. Whilst not as robust as an independent validation set (and potentially more optimistic (Varoquaux, 2017), this still demonstrates the generalisability (and stability) of the predictions. Secondly, the predictions made here are both continuous, predicting a numerical value rather than a label classification, and also non-contemporaneous, we specifically predict long-term outcomes, approximately two-years post MRI/pTBI. Overall, the results seem to suggest a small, yet relatively robust, association between features of this network computed at local (ROI) level and executive function.

Both of these strengths represent difficult, yet vital goals in terms of clinical applicability. Finally, these predictions are independent of many other clinical and demographic variables that may be important in predicting cognitive outcomes post neurological insult. Including such variables, alongside advanced modelling of MRI data, may allow us to recover even greater proportions of variance with our predictive models. Predictive models derived from purely neuroimaging data will only ever account for a proportion of variance (Scheinost, 2019) but an important area for future research will be investigating whether this is above and beyond that accountable for by clinical or demographic variables, to assess the ‘added value’. This was beyond the scope of the current study, due to the additional sample size needed to validate such an approach.

Bootstrapped predictor weightings highlighted that the strongest predictive utility (both positive and negatively weighted) was across fronto-temporal regions. The highest component loadings were across regions of the prefrontal cortex (inferior, superior and medial orbito- frontal gyri) which are particularly important for typical development of executive functions (Fiske and Holmboe, 2019). These regions are frequently reported as different post pTBI compared to controls in terms of regional cortical thickness and volume, indicative of susceptibility to the effects of injury (Wilde, 2011); (Merkley, 2008) ; (Mayer et al., 2015) ; (Wilde, 2012) ; (Dennis, 2017) ; (Dennis, 2016) (see King, Ellis (King, 2019) for a systematic review). Previous work investigating neuroanatomical correlates of the BRIEF-GEC post pTBI found no correlations with mean cortical thickness over multiple ROIs that were chosen a-priori due to their involvement in mediating EFs (Vander Linden, 2019). Our multivariate methodology, however, was able to identify a predictive relationship between *patterns* of morphometric similarity across the cortex and later executive functioning. This suggests that the current methodology replicated previous neuroanatomical differences post-TBI, but also is sensitive to functionally relevant morphometric variation.

Our results show convergence with previous work utilising multivariate methodologies to investigate the neuroanatomical correlates of EF. Previous studies found that, in a child and adolescent cohort, a latent cognitive variable (onto which the BRIEF heavily loaded) was related to voxel-based morphometry of regions similar to those found in the current study, across regions of inferior frontal gyrus, insula, medial orbitofrontal, anterior cingulate cortices (Ziegler, 2013). DTI-derived connectivity of the anterior cingulate and superior frontal regions also distinguished a cluster of children characterised by a profile of elevated inattention, hyperactivity and EF symptoms, who also measured this using the BRIEF (Bathelt, 2018). Only one previous study has investigated the neuroanatomical correlates of executive functions utilising the morphometric similarity approach. He and colleagues (He, 2019) specifically highlighted the role of right medial orbitofrontal cortex as a node of the morphometric similarity network specifically related to predicting inhibitory control performance in healthy adults. Overall, the neuroanatomical specificity of our predictions seems to be plausible in terms of EF maturation, correlates of EF problems in childhood, and consistent with previous investigations using morphometric similarity. However, the current study expands these findings beyond the *meso*-scale architecture of individual regions and suggests that the *meso*-scale organisation of the cortex (indexed using morphometric similarity) in relation to these specific regions is important for subsuming later executive functioning.

EF measurements typically capture information about the performance of cognitive processing skills inherent to EFs, or the functional/behavioural manifestation of executive dysfunction. In the current study, an interesting pattern of findings emerged, along these lines. Specifically, whilst we show that morphometric similarity predicts BRIEF ratings of EF, which measure the functional consequences of executive impairments that manifest in everyday life, the same was not true for directly-measured/performance-based scores. These instead assess the cognitive aspects of executive impairments. Thus, the pattern of results reported here is perhaps unsurprising, given that the BRIEF has been shown to index different components of EF to typical performance-based measures (McCauley, 2010, Ten Eycke and Dewey, 2016) and has been shown to have differential neuroanatomical correlates (for instance (Faridi, 2015) showed differences in neuroanatomical correlations of both performance-based and BRIEF measures of working memory).

There are multiple reasons why, in the current study, morphometric similarity was related to BRIEF and not EF scores. The BRIEF scores index a higher order, functional/behavioural manifestation of executive dysfunction in daily living, beyond that of individual EF skills and that this complexity necessarily requires coordination of brain regions which may be affected in pTBI. Thus, these complex behaviours depend upon distributed networks of brain regions, beyond that of any one specific executive skills that give rise to these higher-order behaviours. Also, in the current study we tried to capture relationships with broad EF outcomes, identifying impairment across different sub-domains of EF for our summary score. Successful prediction of our EF summary score would mean that the pattern of MS represents a ‘core deficit’ in EF performance, which was not the case here. In the absence of a ‘core deficit’ change to brain structure, this could therefore mean that no relationships could be found as there was large heterogeneity in pathological brain changes in line with the heterogeneity in potential EF outcomes in our summary score. However, it is important to note that in a previous study of this cohort we found associations between structural covariance (using cortical thickness alone) and EF performance a) at a using a global measure rather than network-level (King, 2020);

Our study provides novel evidence that the degree to which regions that typically play a role in supporting the acquisition of executive function skills are left-intact is associated with later executive dysfunction following pTBI (Anderson and Catroppa, 2005, Bettcher, 2016, Wilde, 2011). The current study expands these findings, suggesting that, not only is the post-injury structure of these regions prognostically important, but also the relationship of these structures across all areas of the cortex. Nevertheless, there is no current understanding as to the ‘normative’ values for morphometric similarity across the developmental period and thus it is unclear if these inter-individual differences in this metric represent ‘damage’ or ‘abnormalities’ post-injury. Future research should be conducted to properly characterise the developmental trajectories of morphometric similarity across the developmental period.

### 4.3 Predicting EF outcome using individual morphometric features in pTBI

The current study expanded previous studies of neuroanatomical correlates of EF following pTBI by investigating previously unstudied structural features (i.e. curvature) but also using multivariate, rather than univariate approaches to structure. We generated models to predict both EF scores and BRIEF GEC from individual morphometric features however, based upon predicted R^2^ values in the LOO-CV set as a measure of performance, with little success. Interestingly, only Gaussian curvature was able to be used to generate a predictive model of both EF & BRIEF-GEC which had a positive predicted R^2^ value. Cortical volume also had a (small) but positive predicted R^2^ for the LOO-CV model for predicting EF.

We show a difference in the predictive performance of models using morphometric similarity vs. the individual morphometric features used to estimate morphometric similarity. For the BRIEF GEC, only the Guassian curvature single feature model had a positive predicted R^2^. It is important to note, that conducting cross-fold validation on the models which used individual morphometric features as input, revealed increased and potentially better performance in the Gaussian curvature model, compared to the morphometric similarity model. Here, performance increased when tested using 20-fold CV compared to LOO-CV. Whilst this may indicate an improvement to a performance greater than that of the morphometric similarity model, it may also indicate that the Gaussian Curvature model is highly susceptible to outliers in the data, struggling to perform well in outlier cases when they are the only instance being tested on (i.e. in the LOO-CV scenario), resulting in a much reduced R^2^ for the LOO-CV approach. However, across both morphometric similarity and individual feature models, the prediction error (when tested in a LOO-CV sample) was similar in terms of mean absolute error (difference in MAE between models was less than a single point on the original BRIEF scale).

The morphometric similarity model seemed stable when tested under both LOO-CV and cross-fold validation approaches. Whilst the Gaussian curvature model seemed to outperform the morphometric similarity model in cross-fold validation, the conflicting performance compared to the LOO-CV testing indicates the model may be sensitive to specific data points, arguably making it unsuitable for the intended clinical-use case of individual level prediction – outliers are likely to be those with greatest clinical uncertainty or need. Specifically, for predictive models to be of high clinical relevance, robust prediction of outliers (or ‘caseness’) is imperative. Whilst performance of the Gaussian Curvature model in the cross-fold validation testing was more impressive, the model underperforms on additional metrics we used to assess performance (correlation between predicted and actual scores and mean absolute error). Overall, we believe the morphometric similarity model to have performed best among the current tested models.

It is interesting to note that, when we generated bootstrapped CIs of weightings of individual ROIs for the individual feature models, we found that these were wider than those of the morphometric similarity network, suggesting two things. Firstly, the PLS model for individual features is highly unstable and secondly, that there is little regional specificity of damage (as indexed by individual structural MRI features) on the prediction of outcome, especially given the high overlap in CIs between regions. Overall, these findings are first evidence that morphometric similarity may be more sensitive to the clinically relevant structural damage post-neurologic insult, compared to standard structural measures.

These results further support morphometric similarity as a measure of macrostructural organisation of the cortex in assessing the neuroanatomical-basis of cognition. This, and previous studies, assume that this organization is likely to support human cognition. However, in a large scale study of healthy adults (King and Wood, 2020) we did not replicate previous associations with cognitive abilities (Seidlitz, 2018). We believe that, although this organisation may not subsume cognitive functioning *per se*, the disruption of this organisation due to pathology is likely to be symptomatic and result in potential cognitive impairment. In other words, this innate organization is a requirement of healthy cognition, but individual variation in these abilities is explained by other, complex factors (i.e. environmental factors such as SES) and thus variance explained by morphometric similarity is low. However, in pathology/disease, disruption from the typical highly-programmed, *meso*-scale organization of the cortex, as indexed by morphometric similarity, indicates an insult to developing networks and is therefore likely to be a greater contributor to the inability to achieve age-appropriate cognitive performance. This is seen in the relationships between the morphometric similarity network and functioning in this study and other atypical populations (Galdi, et al., 2018, Seidlitz, 2019).

Interestingly, we found Gaussian curvature to be a potential predictor of outcomes in the current study, but not other more ‘traditional’ measures such as cortical thickness or volume. Comparatively, Gaussian curvature, as a measure of brain ‘shape’, has had limited study in terms of TBI (Essex, 2024, Gharehgazlou, 2022, Irimia, 2014). Changes in these geometric properties of the cortical surface may in fact be highly informative; one could imagine a scenario where a local region atrophies (i.e. due to a TBI for instance) but the surrounding tissue remains untouched and so the atrophied region exhibits lower /negative curvature, thus being more concave in nature (Irimia, 2014). Previous work has proposed that geometry changes to the cortical surface, may also represent changes in mechanical tension along axons due to axonal injury (Gharehgazlou, 2022), or even due to deposition of iron-related products of injury (Essex, 2024). A full review of the importance and/or potential of specific measures of cortical surface geometry is beyond the scope of the current investigation, but these findings should inspire future work to investigate their specific value in understanding post-injury outcomes.

### 4.4 Strengths and limitations

One major strength in the current study is that, at the time of writing, it is the first study to investigate morphometric similarity in pTBI. It is also the first study to utilise PLS regression to investigate the neuroanatomical correlates of post-injury functioning in this clinical group. PLS is a powerful exploratory tool for such a task, allowing us to model even collinear or near-linearly dependant predictors as well as generating coefficients that are more stable and easier to interpret than other multivariate approaches such as canonical correlation analysis (Wegelin, 2000). Whilst the LOO-CV approach utilised may lead to unstable estimates of prediction accuracy (Varoquaux, 2017), it is a practical solution to validation of these models where limited sample size is available. It is important to note that the LOO-CV may be over optimistic compared to other schemes (K-cross fold (Varoquaux, 2017), but in comparison to training on the full dataset, it somewhat establishes how the model generalises to unseen data. Therefore, the current results still represent an important step in generating clinically-useful and valid predictive models in pTBI. In the future, greater sample sizes still will be needed to enable less optimistic learning approaches, such as K-cross fold (Varoquaux, 2017).

One potential limitation of the morphometric similarity approach is the evidence here which suggests that edge weights of the network based on morphometric similarity are not reliably estimated. It is important to note that the PLS regression only found models when utilising nodal degree to predict outcome. This binarises the network, thus removing a large amount of information from the network in the form of edge weights. Given that these edge weights did not result in a model with which to predict EF, it may be pertinent to assume that these edge weights contain more noise than predictive signal; potentially as the edge weights themselves cannot be reliably estimated. This may be because the edge weighting is estimated from a correlation between seven morphometric features, a small number of observations with which to reliably estimate a correlation coefficient. This is further evidenced by the high standard deviation of edge weights found by Seidlitz and colleagues (Seidlitz, 2018). However, some edge weighting information will inherently be retained in the binarised network, with retained edges (by definition) being those with a greater edge weight than the edges that were removed from the graph. More reliable estimation of morphometric similarity could result in more accurate predictive models. One potential solution for future research may be to generate/sample a larger number of morphometric, *meso*-scale features from the T1w image with which to estimate morphometric similarity (i.e. (Klein, 2017).

There are also some difficulties in testing the degree to which morphometric similarity predicts EF beyond individual structural MRI features. Whilst our PLS models show that morphometric similarity seemingly contains prognostic information beyond individual features, this is a limited comparison. Morphometric similarity may only lead to greater prediction due to the fact it contains ‘signal’ from all features whilst we compared it a single individual feature at a time. A more sincere comparison for this hypothesis would be to compare the morphometric similarity model against a model that contains all features for all regions. This would allow us to assess whether the covariation structure between these features (as is represented by morphometric similarity) contains greater prognostic information than the features alone. However, this would result in a 7 × 68 predictor matrix, which far outstrips the number of observations we have, and may result in unstable and non-generalizable prediction models due to limited sample sizes. This in fact highlights a potential advantage to the use of morphometric similarity in research settings such as this, in populations where sample sizes are known to be limited (such as pTBI (King, 2019). Morphometric similarity represents a low dimensional representation of the 7 × 68 matrix of individual features, and may in fact contain greater information than the individual features alone, and so is more appropriate for these populations where observation-predictor ratios would otherwise be suboptimal (such as clinical populations where larger sample sizes are harder to obtain).

## 5 Conclusion

The morphometric similarity approach is a methodology that can capture information about the brain’s complex structural organisation. Whilst the amount of variance explained by these predictive models was relatively small, we posit that this methodology is sensitive to the disruption of the highly programmed organisation of the cortex in populations in which the normal developmental trajectories may be divergent. We have expanded the previous univariate investigations of the neuroanatomical correlates of executive functioning (which are inherently limited in detecting the diffuse effects of injury), utilising morphometric similarity and PLS methods which are novel to the field of neuroimaging post pTBI. The current study shows that the complex, *meso*-scale organization of the morphometry of the cortex, namely the morphometric similarity between regions, not only provides additional insights to brain morphometry compared to previous approaches but also possesses the potential to predict more accurately clinically relevant functional outcomes. Predicting EF dysfunction ahead of time, in the clinic environment, will aid in delivering early support to these ‘at-risk’ patients before difficulties result in delays compared to their peers which cannot be caught-up/remediated – this is an important goal for predictive findings such as these.

## Funding

The work in this project was supported by a European Consolidator Fellowship to AGW (PROBIT: 682734). This work was conducted whilst DGK was supported by a studentship from Aston University, School of Life and Health Sciences. This work was supported by a grant from the Victoria Neurotrauma Initiative, Australia (No. CO6E1) to VAA.

## 7 Ethical standards

This study has been approved by the appropriate ethics committee and have therefore been performed in accordance with the ethical standards laid down in the 1964 Declaration of Helsinki and its later amendments. This cohort were originally recruited for a study that had previously received ethical approval via the Human Research and Ethics Committee of Royal Children’s Hospital, Melbourne, Australia. Written informed consent to participate in the original study was provided by the participants' legal guardian/next of kin. A favourable opinion was granted by Aston University Ethics Committee for the secondary analysis of the TBI datasets.

## CRediT authorship contribution statement

**Daniel Griffiths-King:** Writing – original draft, Visualization, Methodology, Investigation, Formal analysis, Data curation, Conceptualization. **Stefano Seri:** Writing – review & editing, Supervision, Funding acquisition. **Cathy Catroppa:** Writing – review & editing, Resources, Data curation. **Vicki A. Anderson:** Writing – review & editing, Resources, Funding acquisition, Data curation. **Amanda G. Wood:** Writing – review & editing, Supervision, Resources, Funding acquisition, Conceptualization.

## Data Availability

Data will be made available on request.
