# Supplementary material for: Network analysis of structural MRI predicts executive function in paediatric traumatic brain injury
Source: Neuroimage Clin. 2024 Oct 9;44:103685. doi: 10.1016/j.nicl.2024.103685 (PMC11531611; doi:10.1016/j.nicl.2024.103685)
Supplement: Supplementary Data 1 [file mmc1.docx]

**Supplementary Materials**

**Differences in graph-level morphometric similarity across ROIs**

When comparing pTBI patients against controls, mean difference in the magnitude of morphometric similarity (adjusted for age at scanning, sex, age*sex, and eTIV) across the brain was not significant following FDR correction, across all network thresholds tested (all p_fdr_ > .05). These can be seen below in Table S1. This was repeated for all ROIs of the unthresholded network to investigate the effect of group on nodal strength, and similar null results were found (all p_fdr_ > .05), which can be seen in Table S2.

| Table S1. Results of GLM to test the effect of group (TBI vs Controls) on average nodal strength, whilst controlling for age at scanning, sex, age*sex, and estimated total intracranial volume (eTIV). | | | | |
| --- | --- | --- | --- | --- |
| Density Threshold | Mean Normalised Strength ^a^ | | *p*_fdr_ ^b^ | Hedges g ^c^ |
|  | Patient | Control |  |  |
| 0.05 | 3.23 | 3.23 | 0.75 | 0.09 |
| 0.1 | 6.25 | 6.25 | 0.98 | 0.01 |
| 0.15 | 9.02 | 9.03 | 0.75 | -0.09 |
| 0.2 | 11.50 | 11.53 | 0.54 | -0.20 |
| 0.25 | 13.64 | 13.71 | 0.37 | -0.29 |
| 0.3 | 15.42 | 15.53 | 0.24 | -0.36 |
| 0.35 | 16.81 | 16.98 | 0.22 | -0.41 |
| 0.4 | 17.81 | 18.01 | 0.22 | -0.44 |
| 1 | -0.20 | -0.13 | 0.54 | -0.19 |
| Note. ^a^ Mean values adjusted for covariates (age, sex, age*sex and eTIV), ^b^ False discovery rate corrected *p* values, ^c^ Corrected for unequal sample sizes | | | | |

| Table S2. Results of GLM to test the effect of group (TBI vs Controls) on nodal strength, whilst controlling for covariates across all ROIs for the unthresholded network | | | | | | |
| --- | --- | --- | --- | --- | --- | --- |
| ROI | *p*_fdr_ ^a^ | Hedges g ^b^ |  | ROI | *p*_fdr_ ^a^ | Hedges g ^b^ |
| lBSTS | 0.46 | -0.47 |  | rBSTS | 0.55 | -0.27 |
| lcACC | 1.00 | 0.00 |  | rcACC | 0.76 | -0.14 |
| lcMFG | 0.94 | 0.04 |  | rcMFG | 0.55 | -0.24 |
| lCUN | 0.46 | 0.39 |  | rCUN | 0.46 | 0.40 |
| lENT | 0.55 | 0.26 |  | rENT | 0.55 | 0.23 |
| lFUS | 0.99 | -0.01 |  | rFUS | 0.76 | -0.15 |
| lIPL | 0.76 | -0.13 |  | rIPL | 0.68 | -0.19 |
| lITG | 0.50 | -0.34 |  | rITG | 0.53 | -0.30 |
| liCC | 0.94 | -0.03 |  | riCC | 0.55 | 0.26 |
| lLOG | 0.90 | -0.07 |  | rLOG | 0.90 | 0.09 |
| lLOF | 0.90 | 0.08 |  | rLOF | 0.94 | -0.03 |
| lLING | 0.55 | 0.26 |  | rLING | 0.51 | 0.32 |
| lMOF | 0.94 | 0.03 |  | rMOF | 0.78 | -0.12 |
| lMTG | 0.46 | -0.39 |  | rMTG | 0.55 | -0.23 |
| lPARH | 1.00 | 0.00 |  | rPARH | 0.55 | -0.30 |
| lparaC | 0.90 | -0.07 |  | rparaC | 0.51 | 0.32 |
| lpOPER | 0.68 | -0.19 |  | rpOPER | 0.78 | 0.12 |
| lpORB | 0.46 | 0.42 |  | rpORB | 0.46 | 0.42 |
| lpTRI | 0.55 | -0.27 |  | rpTRI | 0.55 | 0.28 |
| lperiCAL | 0.70 | 0.17 |  | rperiCAL | 0.51 | 0.32 |
| lpostC | 0.90 | 0.06 |  | rpostC | 0.46 | -0.39 |
| lPCC | 0.90 | 0.09 |  | rPCC | 0.55 | 0.24 |
| lpreC | 0.70 | -0.17 |  | rpreC | 0.50 | -0.36 |
| lPCUN | 0.90 | -0.06 |  | rPCUN | 0.46 | -0.53 |
| lrACC | 0.50 | -0.34 |  | rrACC | 0.90 | 0.06 |
| lrMFG | 0.55 | -0.25 |  | rrMFG | 0.76 | -0.14 |
| lSFG | 0.55 | -0.25 |  | rSFG | 0.55 | -0.23 |
| lSPL | 0.90 | 0.06 |  | rSPL | 0.90 | 0.06 |
| lSTG | 0.50 | -0.36 |  | rSTG | 0.46 | -0.44 |
| lSMAR | 0.94 | -0.03 |  | rSMAR | 0.55 | -0.26 |
| lFP | 0.50 | 0.34 |  | rFP | 0.55 | 0.23 |
| lTP | 0.76 | 0.14 |  | rTP | 0.74 | 0.16 |
| lTT | 0.99 | 0.01 |  | rTT | 0.70 | 0.17 |
| lINS | 0.55 | -0.22 |  | rINS | 0.77 | -0.13 |
| Note. ^a^ False discovery rate corrected *p* values, ^b^ Corrected for unequal sample sizes | | | | | | |

**Exploratory Tests of Differences in graph-level morphometric similarity across multiple groupings**

Given we found no significant differences between patients and controls for morphometric similarity, we post-hoc hypothesised that this may be due to the level inhomogeneous nature of the patient group. Thus we conducted exploratory, post-hoc analyses of potential differences when splitting the patient group based on both outcome and clinical ratings of injury severity.

We firstly compared groups derived from injury severity. Injury severity was derived as described in previous publications of the current dataset, across severities of mild, mild-complex, moderate and severe. In order to reduce multiple comparisons and maintain reasonable group sample sizes, we divided the patients in to a mild group (n= 47) and then a second group comprising all other severities (mild-complex, moderate and severe, n = 36). We then conducted three-way comparisons between each of these groups and controls. We still found no significant differences in graph-level morphometric similarity across all network thresholds (all p_fdr_ > .05). These can be seen in Table S3.

We then divided the patient group based upon those exhibiting clinically relevant cognitive impairment in terms of executive (dys)function at 2 years post-injury. The current study adopted the neuropsychological impairment (NPI) rule proposed by Beauchamp, Brooks, Barrowman, Aglipay, Keightley, Anderson, Yeates, Osmond and Zemek ^1^ has previously been used in regards to TBI ^1-3^. Briefly, performance scores for the neuropsychological test batteries were converted to age-scaled scores (M=10, SD=3) and those assigned to the clinically impaired group were those who performed more than 1SD below average functioning on two or more individual EF measures (EF_poor_, n = 17), whereas those who were impaired on less than two measures were assigned to the without cognitive impairment group (EF_good_, n = 42). We only assigned group membership on the basis of the NPI rule for those cases who had the full battery of EF tests. When comparing morphometric similarity between those with and without impairment and those with controls again, we found no significant differences, and this can be seen in table S4. However, these results may be due to limited power due to the reduction in sample sizes.

| Table S3. Results of GLM to test differences across injury severity in average nodal strength, whilst controlling for age at scanning, sex, age*sex, and estimated total intracranial volume (eTIV). | | | | | | | | | | |
| --- | --- | --- | --- | --- | --- | --- | --- | --- | --- | --- |
| Density Threshold |  | Mean Strength ^a^ | | *p*_fdr_ ^b^ | Hedges g ^c^ |  | Mean Strength ^a^ | | *p*_fdr_ ^b^ | Hedges g ^c^ |
|  |  | Mild  (n=47) | Control  (n=33) |  |  |  | Mod/Sev  (n=36) | Control  (n=33) |  |  |
| 0.05 |  | 3.23 | 3.23 | 0.95 | -0.02 |  | 3.23 | 3.23 | 0.71 | -0.18 |
| 0.1 |  | 6.25 | 6.24 | 0.72 | 0.11 |  | 6.25 | 6.25 | 0.78 | -0.13 |
| 0.15 |  | 9.03 | 9.01 | 0.61 | 0.19 |  | 9.03 | 9.03 | 0.94 | -0.02 |
| 0.2 |  | 11.53 | 11.48 | 0.32 | 0.31 |  | 11.53 | 11.51 | 0.81 | 0.09 |
| 0.25 |  | 13.71 | 13.62 | 0.17 | 0.41 |  | 13.70 | 13.66 | 0.71 | 0.17 |
| 0.3 |  | 15.54 | 15.40 | 0.11 | 0.48 |  | 15.53 | 15.45 | 0.71 | 0.24 |
| 0.35 |  | 16.99 | 16.79 | 0.11 | 0.52 |  | 16.96 | 16.84 | 0.66 | 0.30 |
| 0.4 |  | 18.02 | 17.78 | 0.11 | 0.53 |  | 18.00 | 17.84 | 0.66 | 0.34 |
| 1 |  | -0.11 | -0.17 | 0.70 | 0.14 |  | -0.15 | -0.26 | 0.66 | 0.33 |
| Note. ^a^ Mean values adjusted for covariates (age, sex, age*sex and eTIV), ^b^ False discovery rate corrected *p* values, ^c^ Corrected for unequal sample sizes | | | | | | | | | | |

| Table S4. Results of GLM to test differences across EF impairment groups in average nodal strength, whilst controlling for age at scanning, sex, age*sex, and estimated total intracranial volume (eTIV). | | | | | | | | | | |
| --- | --- | --- | --- | --- | --- | --- | --- | --- | --- | --- |
| Density Threshold |  | Mean Strength ^a^ | | *p*_fdr_ ^b^ | Hedges g ^c^ |  | Mean strength ^a^ | | *p*_fdr_ ^b^ | Hedges g ^c^ |
|  |  | EF_poor_ ^d^  (n=17) | Control  (n=33) |  |  |  | EF_good_ ^d^  (n=42) | Control  (n=33) |  |  |
| 0.05 |  | 3.23 | 3.23 | 0.65 | -0.28 |  | 3.23 | 3.23 | 0.64 | -0.14 |
| 0.1 |  | 6.25 | 6.25 | 0.67 | -0.19 |  | 6.25 | 6.25 | 0.97 | 0.01 |
| 0.15 |  | 9.02 | 9.02 | 0.89 | 0.04 |  | 9.03 | 9.01 | 0.64 | 0.13 |
| 0.2 |  | 11.53 | 11.50 | 0.67 | 0.20 |  | 11.53 | 11.49 | 0.37 | 0.27 |
| 0.25 |  | 13.71 | 13.64 | 0.65 | 0.29 |  | 13.70 | 13.62 | 0.24 | 0.38 |
| 0.3 |  | 15.53 | 15.43 | 0.65 | 0.35 |  | 15.53 | 15.39 | 0.17 | 0.45 |
| 0.35 |  | 16.97 | 16.83 | 0.65 | 0.37 |  | 16.97 | 16.78 | 0.17 | 0.49 |
| 0.4 |  | 18.01 | 17.83 | 0.65 | 0.39 |  | 18.00 | 17.78 | 0.17 | 0.50 |
| 1 |  | -0.13 | -0.09 | 0.78 | -0.12 |  | -0.14 | -0.24 | 0.37 | 0.29 |
| Note. ^a^ Mean values adjusted for covariates (age, sex, age*sex and eTIV), ^b^ False discovery rate corrected *p* values, ^c^ Corrected for unequal sample sizes, ^d^ Based on NPI rule where EF_poor_ represents those with clinically-relevant impairment | | | | | | | | | | |

**Figure S1.
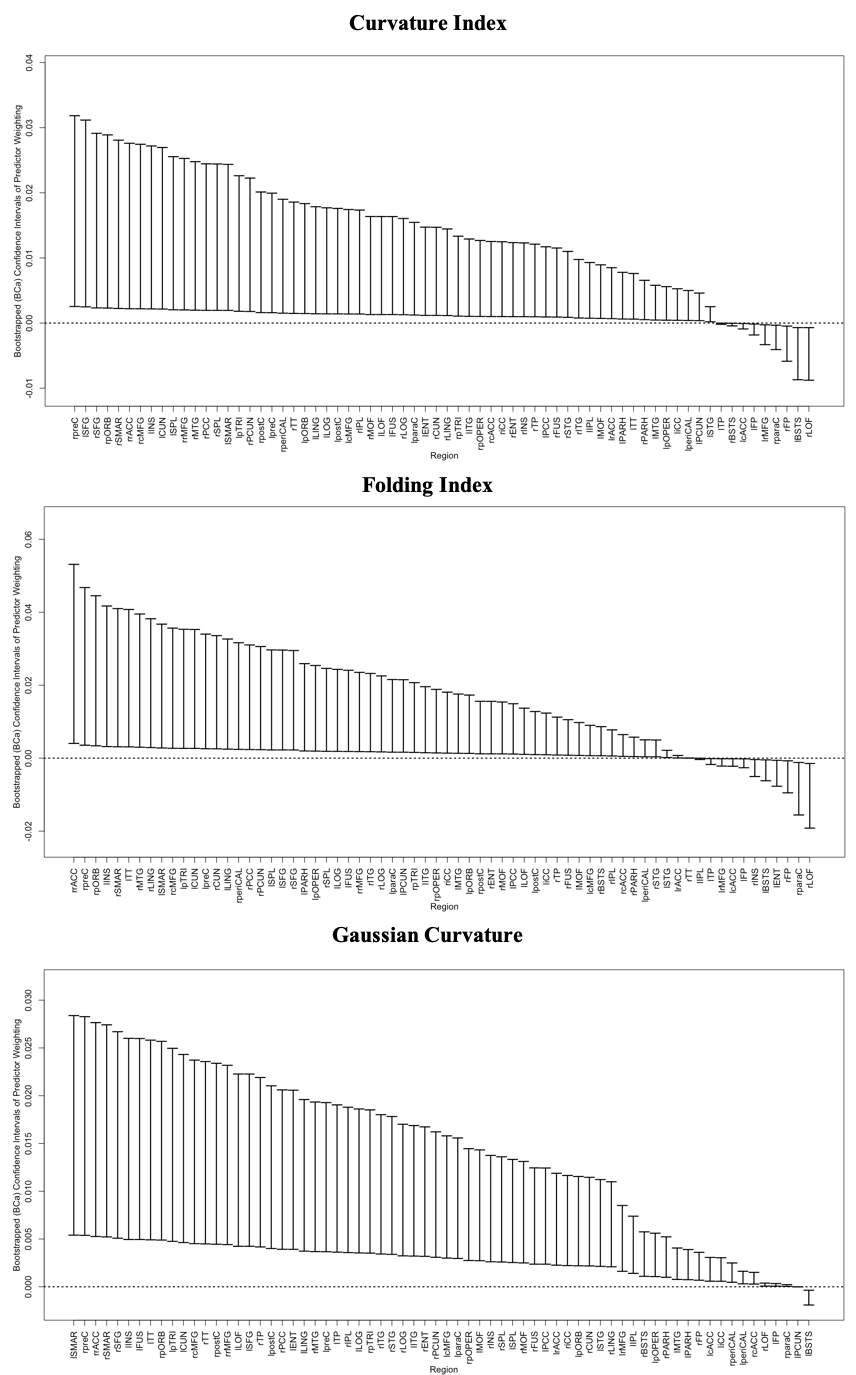

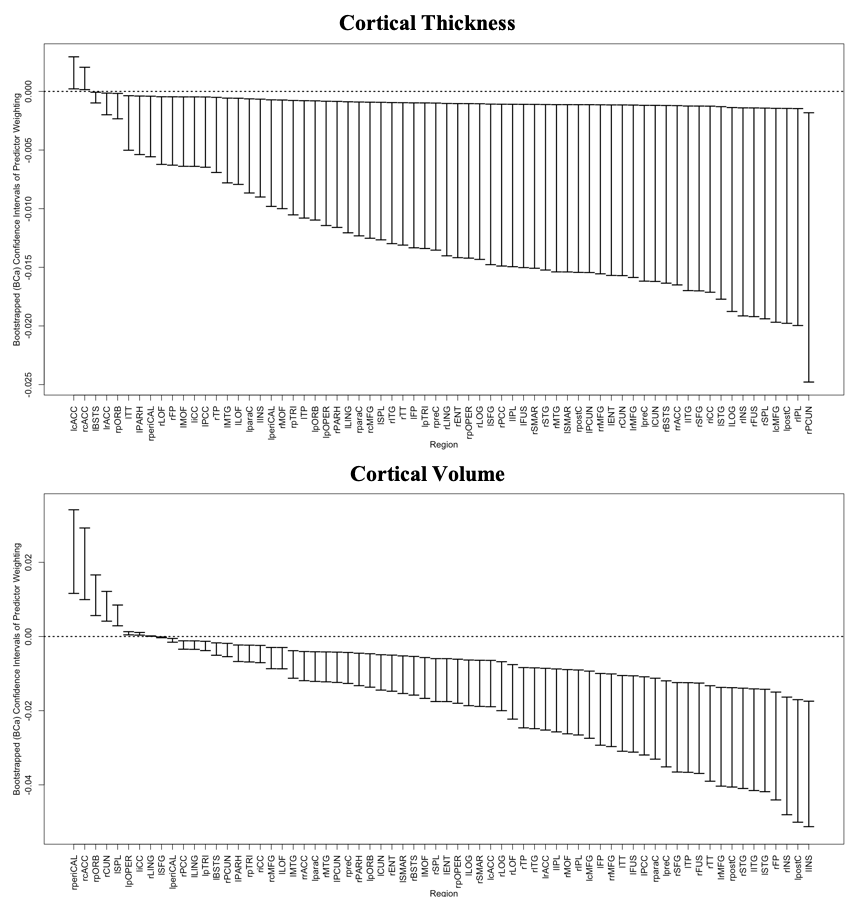
**

Figure S1. The bootstrapped (bias-corrected and accelerated) CI for PLS weightings for each ROI using individual morphometrioc features to predict BRIEF-GEC scores.

**Figure S2.
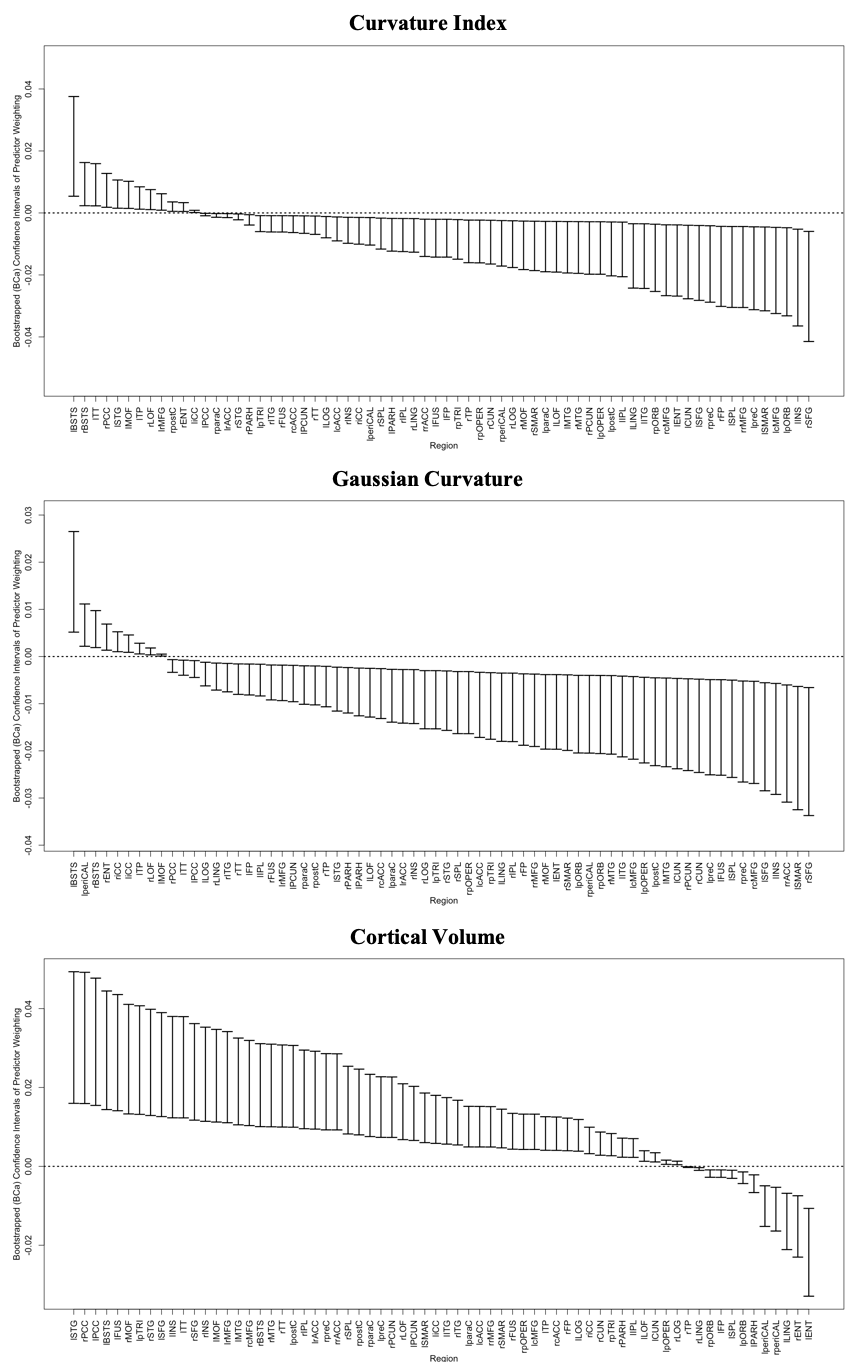
**

Figure S2. The bootstrapped (bias-corrected and accelerated) CI for PLS weightings for each ROI using individual morphometrioc features to predict EF scores.

References

1. Beauchamp MH, Brooks BL, Barrowman N, et al. Empirical Derivation and Validation of a Clinical Case Definition for Neuropsychological Impairment in Children and Adolescents. *J Int Neuropsych Soc*. Sep 2015;21(8):596-609. doi:10.1017/S1355617715000636

2. Beauchamp MH, Aglipay M, Yeates KO, et al. Predictors of neuropsychological outcome after pediatric concussion. *Neuropsychology*. May 2018;32(4):495-508. doi:10.1037/neu0000419

3. Donders J, DeWit C. Parental ratings of daily behavior and child cognitive test performance after pediatric mild traumatic brain injury. *Child neuropsychology : a journal on normal and abnormal development in childhood and adolescence*. Jul 2017;23(5):554-570. doi:10.1080/09297049.2016.1161015
